# The “Slow Horse Racing Effect” on Lung Function in Adult Life in Chronic Obstructive Pulmonary Disease Associated to Biomass Exposure

**DOI:** 10.3389/fmed.2021.700836

**Published:** 2021-07-08

**Authors:** Alejandra Ramírez-Venegas, Francisco Montiel-Lopez, Ramces Falfan-Valencia, Gloria Pérez-Rubio, Raúl H Sansores

**Affiliations:** ^1^Department of Tobacco Smoking and COPD Research, Instituto Nacional de Enfermedades Respiratorias Ismael Cosío Villegas, Mexico City, Mexico; ^2^HLA Laboratory, Instituto Nacional de Enfermedades Respiratorias Ismael Cosío Villegas, Mexico City, Mexico; ^3^Department of Respiratory Medicine, Medica Sur Clinic and Foundation, Mexico City, Mexico

**Keywords:** biomass exposure, tobacco exposure, COPD, early life disadvantages factors, lung function decline, slow horse racing effect, non-smokers

## Abstract

Although different trajectories in lung function decline have been identified in patients with COPD associated to tobacco exposure (TE-COPD), genetic, environmental, and infectious factors affecting lung function throughout life have not been fully elucidated in patients with COPD associated to biomass (BE-COPD). In this review, we present current epidemiological findings and notable advances in the natural history of lung decline in BE-COPD, as well as conditions modeling the FEV_1_ trajectory, such as health insults, during the first years of childhood. Evidence shows that women exposed to biomass smoke reach adult life with a lower FEV_1_ than expected. However, in contrast to the “horse racing effect” predicting an excessive lung-function decline in forthcoming years, as observed in smokers, this decline is slower in non-smokers, and no rapid decliners are observed. Accordingly, BE-COPD might be considered another phenotype of COPD based on assessments of lung function decline. Likewise, other functional and clinical aspects described in this review suggest that this condition might be similar to TE-COPD. More research is needed to fully characterize this subgroup of variants of COPD.

## Introduction

As an heterogenous disease, chronic obstructive pulmonary disease (COPD) has diverse clinical presentations, such as symptoms, exacerbations, comorbidities, quality of life and also different lung function trajectories (1). Currently, it is well-recognized that COPD is not just a single entity and patients presenting airflow limitation may exhibit different COPD phenotypes, such as inceptions, early stage evolution and progression may be quite different (2, 3). Natural history of COPD has evolved regarding the concept as an isolated disease. In this sense, recent evidence shows that the disease, in terms of clinical characteristics and lung function, is much more heterogeneous than previously thought (4–6). COPD encompasses a complex and heterogeneous group of disorders resulting from different risk factors that lead to different clinical courses and therefore different natural histories of the disease (7–9), such as COPD associated to biomass smoke (BE-COPD) (10, 11).

Regarding lung function decline, patients with COPD may have at least four different trajectories in their decline, which are closely related to genetic, environmental, and infectious factors that impact their lung growth. Thus, the lung function of an individual with COPD can be determined by factors that influence lung growth in pregnancy and the childhood period (12, 13).

Regarding the natural history of COPD due to biomass, this trajectory of lung function is less clear throughout life. Data on the trajectory of lung function for a long time in BE-COPD are only available from a single published cohort study (14). Similarly, research on lung function in rural children exposed to biomass is also limited (15, 16).

Many questions regarding BE-COPD remain. For example, the mechanisms associated with the different behaviors of lung decline of these patients in comparison with COPD associated to tobacco smoke (TE-COPD) as well as the influence on lung growth during childhood in the trajectory of decline in lung function are unclear.

Because the socioeconomic context is crucial in the natural history of COPD associated with biomass, we first analyze the epidemiology of biomass exposure and their implications in terms of the natural history of the disease in this review. Second, we highlight the trajectory of lung function decline in exposed women and showed that it starts in childhood and continues to adult life. Additionally, differences and similarities among TE-COPD and BE-COPD lung function trajectories are discussed.

### Epidemiology of the Differences Between Biomass and Tobacco Exposure

Although in epidemiological terms, the main difference between biomass and tobacco exposure and COPD is related to the social context, both of them are, strictly speaking, biomass products. However, smoke coming from tobacco cigarettes is highly processed and industrialized with a lot of inorganic toxic compounds. On opposite to biomass smoke coming from unprocessed organic matter, most of information about COPD related to tobacco is based on manufactured cigarette smoke. The tobacco epidemic developed as a result of innovations in the tobacco industry between 1913 and 1920. Then, during the 60s, the cigarette industry experienced unregulated growth in the United States and worldwide (17), killing thousands of millions of people. Exposure to biomass has occurred since manhood began to use organic fuel materials for cooking and heating, affecting billions of women and children (18) who were not aware of the impact of exposure on the health of exposed individuals for centuries (7, 19).

Although tobacco smoking for recreational purposes was driven by the tobacco industry, creating individuals with a need for nicotine (20), the use of biomass was a necessity for women in rural areas to subsist, cook their food and heat their households because poverty prevented access to electricity (21). Although tobacco smoking was symbolically associated with glamor and economic development, in more recent decades the prevalence of tobacco smoking and BE-COPD in terms of the socioeconomic status, affects similarly low-middle income groups (22). Unfortunately, biomass smoke has always been associated with poverty and the lack of development.

The World Health Organization (23) has estimated that ~1,100 billion people globally have been exposed to tobacco. These figures are notably lower than those reported for biomass exposure (three billion people) which is approximately half of the population around the world. This proportion is higher in developing countries and especially in rural areas (24).

#### Biomass in Rural Households Causes COPD in Women

Rural women around the world have their leading role mainly inside households and spend must part of the day in homework and cooking activities, whereas men for different reasons are away from home. Traditionally, women are exposed to pollution inside home where worldwide dominates the list of high levels of exposure to various pollutants, especially in developing countries. Due to low combustion efficiency of biomass fuel, gaseous pollutants that are very harmful to respiratory health, such as carbon monoxide, hydrocarbon and chlorinated organic, are generated (25); this type of household pollution is one of the main causes of BE-COPD. Women exposed to high levels of indoor smoke, especially in low-income countries, have two-fold risk to suffer from COPD than women who do not (26). As consequence, globally in 2019, COPD was the 3^rd^ cause of death, and it was responsible for 6% of the total deaths. According to WHO, COPD was the 4^th^ cause of death in lower-middle-income countries in 2019 addressing more than 1 million deaths (27).

The evidence of COPD associated to biomass exposure is overwhelming. Women exposed to indoor smoke are more likely to develop chronic bronchitis than women who cook with other sources of energy (electricity or gas) (28). As a matter of fact, it may be considered a disease almost exclusively for women because the prevalence, comparatively in men, is much higher (29). In this sense, it may be a double public health issue; first of all because affects only women, and secondly because of the worldwide growing number of ill women (30).

#### The Beginning of the Imperceptible Airway and Lung Damage in Biomass Exposure

In rural areas, it is very common that women have prolonged periods of cooking with a high smoke exposure per day; this exposure occurs indoors in unvented places with open fires that operate at low temperatures. This method of cooking produces a variety of air pollutants. The levels of pollutants inside homes burning biomass in unvented open fires are extremely high, that is, in the milligram per cubic meter range (31).

Women are exposed to biomass smoke at all stages of life, that is, during pregnancy and childhood and especially in adolescence when they begin to cook. Women and girls receiving the largest cumulative exposures are often exposed throughout their lives. They spend an average of 4 to 8 h daily in the kitchen, usually in an enclosed space with poor ventilation. They spend more than half of their waking hours in the kitchen, usually in a very limited unventilated space. This exposure may represent 40 years of their entire life. The total time of exposure is about 60,000 h during which they inhale more than 25 million liters of particles contained in the smoke (32). This chronic and high-level exposure predisposes patients to chronic inflammation at the airway walls, and such damage may take many years to become apparent. During childhood, the consequence of this persistent and prolonged biomass exposure may produce repeated respiratory infections that may remodel the structure of the airway walls and predispose an airway-predominant COPD phenotype as adults (33).

### Natural History of BE-COPD

The time course concept of the natural history of COPD has radically changed. In the traditional concept, susceptible individuals who smoked started to experience a decline in lung function in an accelerated manner after 40 years (34). However, recent data from cohorts (35, 36) of smokers suggest that the beginning of COPD is not a clearly defined concept (37). It seems that the natural history of this disease starts at the moment of exposure to a certain causative agent in a susceptible person (13). The age of onset of biomass exposure begins earlier in life (*in utero* and from the neonatal period) compared with that noted in smokers whose onset of smoking occurs several years later, usually during the adolescence years (15, 38). During biomass exposure, young children and infants are also exposed to very high levels of particle matter from biomass smoke (21) because are carried on their mother's back most of the day. Chronic aggression beginning in childhood may act as an early challenge to airway structure (39). The early years of life are crucial because exposure can affect lung function during adult life. Children exposed to biomass experience more acute respiratory infections, pneumonia and asthma than children without that exposure (40). Regarding tobacco smoking, it is well-known that maternal smoking is particularly harmful when young children are exposed to smoke during the first phase in early life in which are more vulnerable (38). Accordingly, in a cohort of children reported by Svanes et al. (36), these events were called “early life disadvantages factors” and cause lung function not to reach the maximum level of growth compared with children without such exposures (36, 41). These early life events might lead to early COPD. The impact of early risk factors has been investigated exclusively in smokers. We assume that similar to smokers, children exposed to biomass present more risk factors that will influence the lung growth that predisposes them to develop COPD.

#### COPD Time Course From Childhood to Adult Life in Response to Biomass Exposure

**Figure 1:**
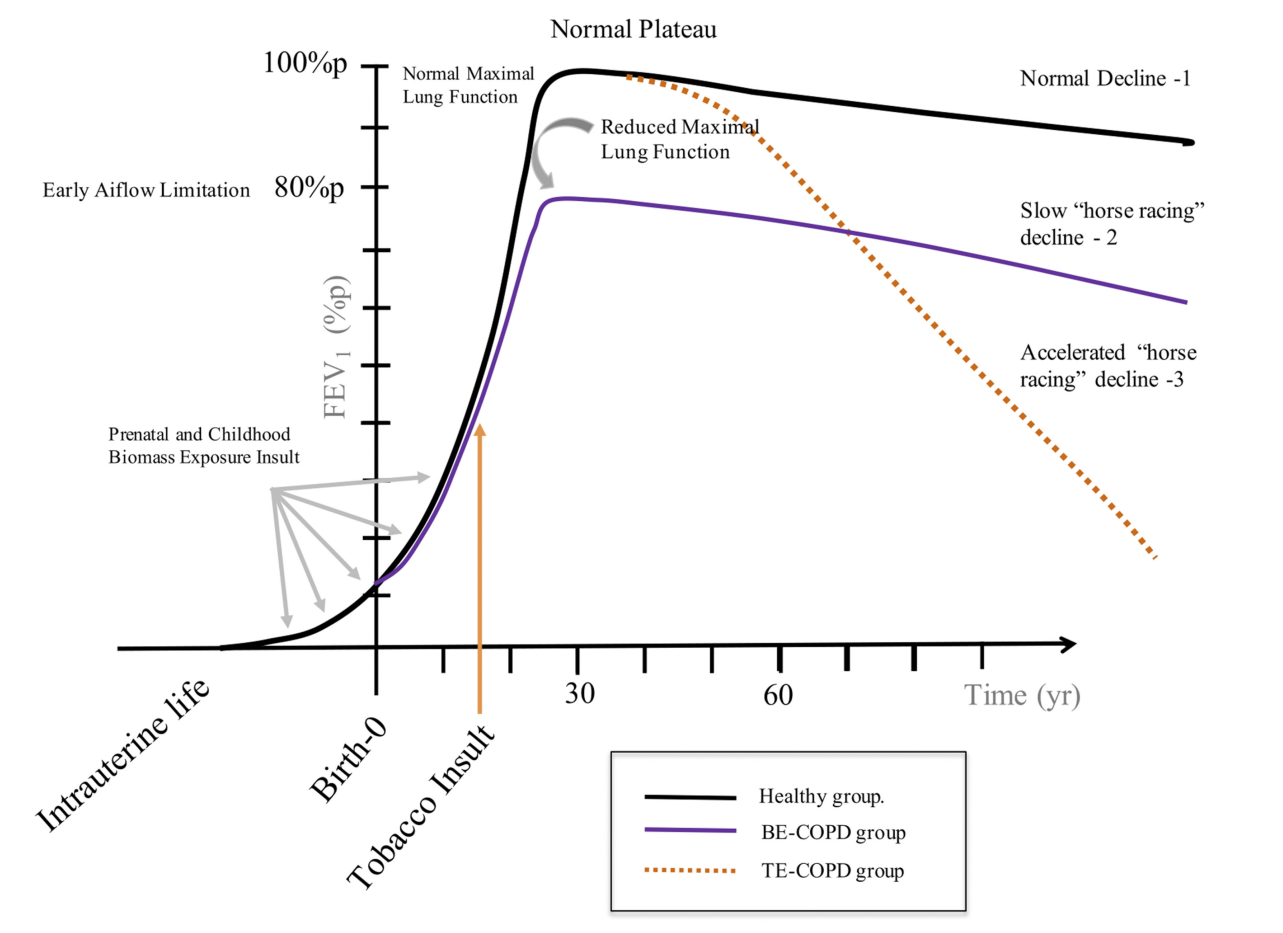
This figure exposes three different trajectories of lung function. Trajectory 1 (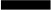) shows a group of healthy individuals with no history of insults during childhood nor through all of them life, who had a normal maximal lung function age of 25 and had a normal decline in lung function. Trajectory 2 (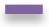) shows a group of women who had history of exposure to biomass smoke both in the prenatal and childhood phases, with adverse event during childhood, who had: (a) a reduced maximal lung function (around 80% of predicted) and showing a slow decline after the plateau but with similar trajectory as a normal group. Trajectory 3 (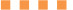) shows the cohort of individual who became smokers at the adolescence, they shape a similar curve as healthy individuals reaching a normal maximal lung function. However, after plateau an accelerated decline is observed.

There are no available data to evaluate lung growth from childhood to adult life in a population exposed to biomass since the childhood period. Recently, Heinzerling et al. (15) compared annual changes in spirometry parameters in 3 groups of children aged 5 years. The home of the first group, which was the control children's group, was provided with a clean stove intervention since pregnancy to prevent household air pollution (HAP) from solid fuel combustion; the second group received a clean stove after 18 months of birth, and the third group received a clean stove when they were 5 years old. Their results showed that limitations in lung function during childhood might start at birth. A decreasing trend of 44 ml per year in the FEV_1_ growth rate in children who did not receive a clean stove was noted compared with children who received a clean stove. The clinical implications of this study are relevant for the consequences of lung function in adult life. If we transpose these findings to susceptible women aged 25 or 30 who are exposed to biomass smoke, normal lung function over the following years would never be reached given the cumulative loss of 880 ml in FEV_1_. Therefore, in adult life, women begin their FEV_1_ plateau phase at a lower level compared with women without biomass exposure (Figure 1). Other studies have assessed the impact of biomass exposure on lung function growth in children (16, 42). Malawi children non-exposed to domestic air pollution had lower carboxyhemoglobin levels and higher FVC than controls. However, no changes with FEV_1_ were found (16).

#### The “Slow Horse Racing Effect” of Lung Function in Adults With Biomass Exposure

The maximal growth development in lung function is reached at approximately 25 years of age, remains constant for a period of 10 years and then slowly begins to decrease 25–30 ml annually in healthy people who have never smoked (43, 44). In women exposed to biomass, we do not know how long the plateau phase lasts. According to the paragraph above and Figure 1, it would be expected that women with BE-COPD start their lung function decline from a lower FEV_1_ than normal women or women with TE-COPD; therefore, these women should exhibit a significantly greater reduction in FEV_1_ than smokers. Nevertheless, this is not the case. It seems to be that women exposed to biomass smoke reaching adult life with a lower FEV_1_ level do not show excessive lung function decline in forthcoming years, as observed in smokers with rapid decline (45).

**Figure 2:**
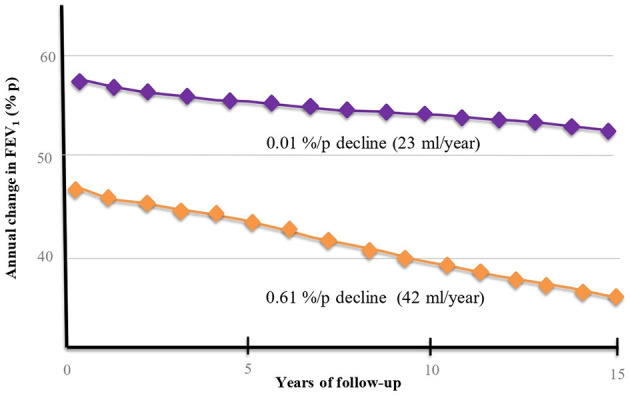
FEV_1_ decline over time in BE-COPD group (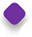) and TE-COPD (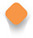) group. The Y axis shows annual change of FEV_1_ as percent of predicted (%/p), whereas X axis shows FEV_1_ decline through 15 years of follow up. In BE- COPD group the FEV_1_ starts at higher values (%/p) than TE-COPD group. In order to compare the decline between groups we also show absolute values (ml/year). The annual decline in absolute values in TE-COPD group is two-fold faster than BE-COPD (42 vs. 23 ml/year).

**Figure 3:**
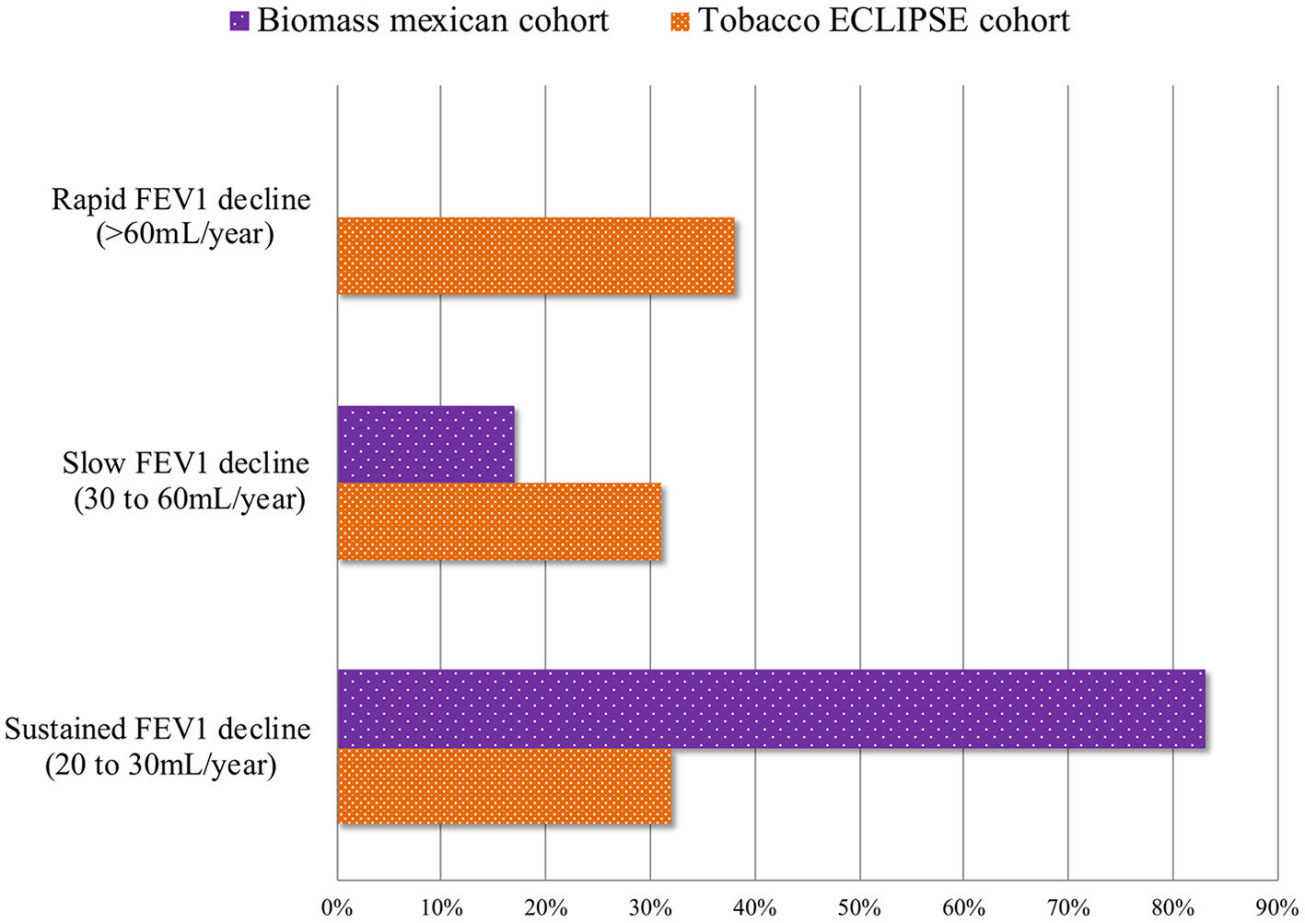
This figure shows the contrast of the lung function decline according to the prevalent phenotype. Rapid decliners phenotype: lung function decline >60 ml/year; slow decliners phenotype: lung function decline 30 to 60 ml/year; sustained decliners phenotype: lung function decline 30 ml/year up to 20 ml/year. Proportion of rapid decliners is higher in TE-COPD in comparison to BE-COPD group who have the largest group of sustained decliners and less proportion of slow decliners.

The severity in lung function and its progression in FEV_1_ decline in BE-COPD, have a different behavior compared with that observed in smokers. The largest cohort of BE-COPD (14) patients with a large period of follow-up has helped us to better understand the functional course of the disease (14). Three characteristics of FEV_1_ decline were consistently observed in the Mexican cohort of non-smoking women. (1) Slower decline: Accordingly, the time-course behavior of FEV_1_ decline of patients with COPD associated with biomass (14) was significantly slower that caused by tobacco exposure. In the Mexican cohort, the annual rate of decline was two-folds higher for the smokers group compared with the non-smokers group (42 vs. 23 ml, respectively, *P* < 0.001) (Figure 2). Similar lung function behavior was recently shown by Salvi et al. (46); however, the follow-up time in this study was only 2 years. In this cohort, the annual decrease in FEV_1_ was 130 ml in smokers and 80 ml in non-smokers. This pattern of slow decline in lung function in women exposed to biomass may be identified as “the slow horse racing effect” in contrast with the rapid pattern of decline in smokers, which was previously recognized in the 1980s (47) as a horse racing effect (Figure 1). (2) Absence of rapid decliners: The proportion of rapid decliners in subjects with BE-COPD is minimal or non-existent (1%). Compared with other international COPD cohorts (5, 48), there are no faster or rapid decliners in BE-COPD, whereas up to 38% of smokers may be rapid decliners in the Eclipse cohort (5, 14). In contrast, 83% of women with BE-COPD are sustained decliners, only 32% of patients in the Eclipse cohort and 25% in the Nishimura cohort (5, 48) are sustained decliners. The label of sustained decliners is based in the concept that Nishimura et al. introduced according on the magnitude of annual change in post-bronchodilator FEV_1_. They introduced the following three percentile levels: annual change less than the 25th percentile means rapid decliners; between 25th percentile to 75th percentile means slow decliners; and greater than the 75th percentile means sustained decliners (48). In our results, a percentile greater than 75th in the sustainers BE-COPD group was <30 ml/year, which is consistent with the amount of ml FEV_1_ decreased annually in healthy non-smokers, which on average is 30 ml after 35 years old (44) (Figure 3). (3) Less intravariability in lung function decline: In comparison with smokers, BE-COPD lung function shows less heterogenicity and intravariability in the annual change in the lung function rate. For instance, different smoker COPD cohorts (5, 48, 49) show greater variability in FEV_1_ decline, ranging from −180 to 100 ml, whereas the variability ranged from −40 to 10 ml in women with BE-COPD. This is strong evidence that at least the disease in BE-COPD is more homogeneous in terms of lung function decline.

## Discussion

The “slow horse racing effect” of the FEV_1_ slope in these women may be explained in part by the following characteristics: (a) The absence of emphysema in this population. Different studies have demonstrated the limited presence of emphysema measured by computed tomography (CT) (50, 51) or by autopsy (52). One explanation regarding the absence of emphysema could be related to the respiratory pattern when women inhaled biomass smoke. The type of inhalation pattern that these women have during cooking probably prevents the damage from spreading beyond the small airway, leading to an airway-predominant COPD phenotype (50, 53). In recent years, different studies have demonstrated that small airway disease is mainly affected in this population (50, 53). On the contrary, the inhalation pattern use by smokers causes the particles of cigarette smoke to settle deep into the lungs. In the first phase, the smoke is inhaled into the mouth where it is held, after a pause, in the second phase, an additional volume of inhaling air causes that smoke to enter deeply into the lungs. This larger inhalation volume in smokers allows the smoke from the inhaled cigarette to increase its deposition in the lung parenchyma and leading over time the presence of emphysema-predominant-COPD phenotype. In biomass exposure, this type of deep inhalation does not occur (54). These findings were recently validated by Nicolaou et al., by using a computational geometry models of the upper and lower airways, they demonstrated that particle matter of smoke is deposited in higher doses in the lungs compared to biomass smoke, which may explain the difference in the two phenotypes (55). In addition, a peculiar pattern of vulnerability and susceptibility in female smokers may predispose more frequently to emphysema in comparison to women exposed to biomass (56). Different studies have shown a strong relationship between emphysema measured by CT and FEV_1_ decline (48). (b) Burrow's theory: Since the paradigmatic observations by Burrows and colleagues in 1986 (47) about the longitudinal spirometric changes in smokers, the so-called “horse-racing effect” has suggested that a low FEV_1_ predicts a faster decline in FEV_1_ and as a consequence the development of COPD. This effect was only seen in male smokers, but these results were not observed in smoking women. Currently, this effect is not homogeneous, and the FEV_1_ slope is not similar in smokers. A new analysis and new variables, such as height, need to be considered in a new model to predict FEV_1_ decline in women. This information is particularly relevant in the Mexican cohort of women whose average height is 148 cm, which is notably lower than smokers (men and women) studied by Burrows. In this sense, studies performed in women exposed to biomass showed that genetic susceptibility (57–60) together with the socioeconomic context may influence growth and decrease lung function, as previously demonstrated by some reports (61–63).

Accordingly, BE-COPD might be considered another phenotype of COPD based on assessments of lung function decline. Likewise, other functional and clinical aspects suggest that this condition may resemble to TE-COPD. Different cross-sectional studies comparing the clinical and functional profile have highlighted that women exposed to biomass smoke persistently show more phlegm (50, 64, 65), wheezing (64, 65), and chronic bronchitis (32, 66); similar or more dyspnea (50, 53, 67) and quality of life affection (46, 50, 68, 69); similar frequency of exacerbations (70, 71), similar exercise capacity (50, 67, 69), and more hypoxemia (50, 67, 72) than smokers. Moreover, mortality also is similar to that observed in smokers (72, 73), although this issue has conflicting results from only two studies with opposite conclusions. Therefore, the rate of mortality it is not fully concluded because of this observational controversy (see Table 1).

**Table 1:** COPD characteristics of subjects exposed to biomass and tobacco smoke.

	**Biomass**	**Tobacco**	**References**
**Clinical characteristics**			
Age of awareness of respiratory symptoms	>65 years old	50–60 years old	(50, 64, 65)
Wheezing	++	+	
Phlegm	++	+	
Chronic bronchitis	++	+	(32, 66)
Dyspnea^*^	+++	+++/++	(50, 53, 66)
Affection of quality of life^*^	+++	+++/++	(46, 50, 68, 69)
Exacerbation frequency	Similar as tobacco (++)	Similar as biomass (++)	(70, 71)
Walking distance (6 MWD)	Similar as smokers	Similar as biomass	(50, 67, 69)
Hypoxemia	++	+	
SpO_2_ at rest	Lower than tobacco (++)	Low (+)	(72)
**Lung function**			
Airflow obstruction	Mild to moderate	Moderate to Severe	(50, 73–75)
DL_co_	Normal	Low	(65, 68, 76)
Bronchial Hyperresponsiveness	++	+	(77)
Annual lung function decline	Sustained decline/non-faster decline	Less sustained than biomass	(14, 46)
**Small airway affection**			
Small airway resistances	Extremely affected	Affected	(46, 50)
Computed tomography findings	Airway thickening Air trapping pattern Without emphysema	Emphysema predominant. Less air trapping.	(51, 78)
Pathology pattern	More anthracosis, Fibrosis peribronquiolar Pulmonary arteriole intimal thickening	More emphysema	(52, 53)
**Mortality**			
Unadjusted by lung function	Better than tobacco	Worse than biomass	(72, 73)
Adjusted by lung function	Similar as tobacco	Similar as biomass	

*+++, more frequent or more affected; ++, very common; +, common*.

* *Measured with different instruments. 6 MWD, 6-min walking distance; PaO_2_, arterial oxygen tension; SpO_2_, arterial oxygen saturation by pulse oximetry; DL_CO_, carbon monoxide diffusing capacity*.

## Limitations

BE exposure is a “new-old disease” having therefore very new insights coming from very new and updated information about a chronic and ancient pollutants exposure. Unfortunately, information on the longitudinal behavior of lung function in BE-COPD is scarce and conclusions cannot be globally considered. For instance, limitations related to this review focused on FEV_1_ decline are mostly derived from the small amount of original articles in comparison with the vast number of papers on COPD associated to tobacco smoking. Research of lung decline on BE-COPD has come particularly from our group (14) and one research come from India (46). Furthermore, it has not been possible to unify diverse aspects of BE-COPD such as strategies to quantify the extent of exposure, type of fuel (wood, crops, etc.) and time of follow-up. In this sense, the statements of this review are limited to wood smoke which is the most common matter used by the patients that we reported (14, 72) and therefore is not possible to generalize our assertions. Multicentric studies including BE-COPD cohorts in different developing countries around the world need to be studied with the same criteria, including same form to evaluate exposure, symptoms, CT, and standardize lung function assessment in order to confirm our assertions.

## Strengths

The large majority of issues and insights in this review were not straightly addressed and discussed in the original publications (14, 72). This review allows to look at BE-COPD as a phenotype of COPD leading to researchers to speculate on different issues of this disease such as the causes of the variety of trajectories of FEV_1_ in women exposed to biomass smoke. Probably the most appealing strength of this review is the solid source of information of one cohort with prolonged followed-up for 15 years with at least three measurements of lung function during follow-up that allowed to perform an individual slope for each patient necessary to measure the real decline in lung function. As far as we know, there are no other BE-COPD cohorts having had these characteristics nor such a long period of follow-up on lung function.

## Conclusion

BE-COPD is a disorder clearly associated with poverty and sociocultural aspects that directly impact the lung growth of these women. This entity might be regarded as a clinical subgroup or “variant” of COPD. More research is needed to understand the role among earlier exposure to biomass smoke, genetic predisposition and sociocultural and economic factors leading to the slow but undeniable reduction in FEV_1_ in women exposed to biomass smoke. Presently, it is clear that the trajectory of lung function decline maintains a close relationship with early risk factors during pregnancy and childhood leading to BE-COPD.

## Author Contributions

AR-V and RS contributed to the conception, design, and first draft of the manuscript. FM-L, GP-R, and RF-V contributed equally to the edition and revision of the manuscript. AR-V, RS, and FM-L contributed equally to manuscript revision and approved the submitted version. All authors contributed to the article and approved the submitted version.

## Conflict of Interest

The authors declare that the research was conducted in the absence of any commercial or financial relationships that could be construed as a potential conflict of interest.
